# Bio-Based *Eucommia ulmoides* Gum Composites with High Electromagnetic Interference Shielding Performance

**DOI:** 10.3390/polym14050970

**Published:** 2022-02-28

**Authors:** Hailan Kang, Sen Luo, Hongyang Du, Lishuo Han, Donghan Li, Long Li, Qinghong Fang

**Affiliations:** 1 College of Materials Science and Engineering, Shenyang University of Chemical Technology, Shenyang 110142, China; kanghailan@syuct.edu.cn (H.K.); luosen971012@163.com (S.L.); 13940204549@163.com (H.D.); hanls0022@163.com (L.H.); lidonghansyuct@126.com (D.L.); 2 Key Laboratory for Rubber Elastomer of Liaoning Province, Shenyang University of Chemical Technology, Shenyang 110142, China

**Keywords:** *Eucommia ulmoides* gum, carbon nanotubes, graphene, electromagnetic shielding

## Abstract

Herein, high-performance electromagnetic interference (EMI) shielding bio-based composites were prepared by using EUG (*Eucommia ulmoides* gum) with a crystalline structure as the matrix and carbon nanotube (CNT)/graphene nanoplatelet (GNP) hybrids as the conductive fillers. The morphology of the CNT/GNP hybrids in the CNT/GNP/EUG composites showed the uniform distribution of CNTs and GNPs in EUG, forming a denser filler network, which afforded improved conductivity and EMI shielding effect compared with pure EUG. Accordingly, EMI shielding effectiveness values of the CNT/GNP/EUG composites reached 42 dB in the X-band frequency range, meeting the EMI shielding requirements for commercial products. Electromagnetic waves were mainly absorbed via conduction losses, multiple reflections from interfaces and interfacial dipole relaxation losses. Moreover, the CNT/GNP/EUG composites exhibited attractive mechanical properties and high thermal stability. The combination of excellent EMI shielding performance and attractive mechanical properties render the as-prepared CNT/GNP/EUG composites attractive candidates for various applications.

## 1. Introduction

With the widespread application of advanced electronic devices, our lives are enhanced by ever greater convenience. However, the development of electronic devices has created considerable quantities of electromagnetic (EM) pollution due to electromagnetic radiation and EM interference; this pollution affects human health and the normal operation of other devices [1,2,3,4,5,6]. In order to avoid these issues, many novel materials with high EMI shielding performance were explored with the aim of addressing problems related to EMI and radiation [7]. Conductive polymer composites (CPCs) consisting of polymer and conductive fillers were explored as promising alternative EMI shielding materials; these materials are generally metal-based materials [8,9,10,11,12]. Research on CPCs was motivated by their low weight, flexibility, resistance to corrosion and good processability [13,14,15,16].

Numerous efforts were devoted to the design and construction of CPCs using compounding polymers with carbon-based nanofillers, such as carbon black (CB), carbon fibers (CFs), carbon nanotubes (CNTs) and graphene [17,18,19,20,21,22,23]. For example, Cheng et al. [24] prepared CPCs using an ultrahigh-molecular-weight polyethylene/polypropylene (PP) blend with conductive CB, which exhibits an absorption-dominated EMI shielding effectiveness (EMI SE) as high as 27.29 dB over the X-band frequency range. Lin et al. [25] developed CF/PP composites with an EMI SE of ~20 dB via adding 15 wt% CFs. Since both CNTs and graphene have exceptional mechanical, electric transport properties and extremely high aspect ratios, they make excellent conducting fillers in CPCs for EMI shielding applications [26,27,28,29,30,31]. Wu et al. [32] prepared CNT/PP composites with an EMI SE of 43.1 dB by adding 5 wt% CNTs. Abraham et al. [33] produced CNT/styrene-butadiene rubber composites with ionic liquids, and the EMI SE of the composites was found to reach 35.06 dB at 18 GHz when adding 10 wt% CNTs. Lu et al. [34] fabricated graphene nanoplatelet (GnP)/ethylene propylene diene monomer rubber (EPDM) composites via a combination process that included mixing, ultrasonication and compression. The GnP/EPDM composites exhibit a high EMI SE of 33 dB in the range 8.2–12.4 GHz and 35 dB in the range 12.4–18 GHz. 

The EMI shielding performance of CPCs is heavily dependent on the conductivity, dispersion and connectivity of fillers in the matrix [35,36]. Three-dimensional (3D) hybrid filler materials were successfully constructed via the hybridization of one-dimensional (1D) CNTs with two-dimensional (2D) graphene, which can overcome the problem of dispersion faced by the individual fillers. The π–π interaction between the CNTs and the graphene is beneficial in the nanoscale separation of the individual CNTs and graphene sheets [37,38]. Moreover, the long CNTs were found to be entangled with the graphene nanosheet, which inhibited their aggregation and provided efficient pathways for electron transfer. Zhao et al. [39] produced a material with an improved EMI shielding performance using polydimethylsiloxane with CNT/graphene hybrids; this material achieved an EMI SE of 31 dB. Kim et al. [40] fabricated CNT/GNP/PP composites with an EMI SE of 36.5 dB at 1.25 GHz. Mohammed et al. [41] prepared PP/polyethylene composites using GNP/CNT hybrid nanofillers. Across this research, it can be seen that using CNTs and graphene as hybrid fillers yield an effective route to the fabrication of CPCs with excellent EMI shielding performance. 

With concern regarding the price fluctuations of fossil fuel reserves and the increasing environmental awareness, eco-friendly polymeric materials have attracted increasing research attention. *Eucommia ulmoides* gum (trans-1,4-polyisoprene, EUG) is extracted from the *Eucommia ulmoides* Oliv and an isomer of natural rubber. Due to its unique dual nature combining properties of both plastics and rubber, EUG was used as a thermoplastic material, thermoelastic shape memory material and as a highly elastic material in many fields. To date, there is little research regarding the use of EUG as an EMI shielding matrix. In the present work, we used EUG with a crystalline structure as a matrix material and CNT/GNP hybrids as conductive fillers to construct a shielding composite with a high EMI shielding performance. The effect of the CNT/GNP hybrids on the morphology, electrical conductivity and EMI shielding properties was investigated. Furthermore, the EMI shielding mechanism of the CNT/GNP/EUG composites was analyzed.

## 2. Materials and Methods

### 2.1. Materials

*Eucommia ulmoides* gum (EUG) was supplied by Shandong Beilong Eucommia Chemical Industry Co., Ltd., Weifang, China. Carbon nanotubes (CNTs, with a diameter in the range of 8–15 nm and length of 1–10 μm) were purchased from the Shanghai Kajite Chemical Technology Co., Ltd., Shanghai, China. Graphene nanosheets (GNPs) were provided by Shenzhen Hongda Changjin Technology Co., Ltd., Shenzhen, China. Triton X-100 (polyethylene glycol tertoctylphenyl ether) used in this work was provided by Shanghai Youdao Aoba Chemical Co., Ltd., Shanghai, China. 

### 2.2. Methods

#### 2.2.1. Preparation

CNTs and GNPs were modified via the use of Triton X-100. An amount of 0.1 g of GNPs (or CNTs) was mixed with 1 mL of Triton X-100 and subsequently grounded manually in an agate bowl for 30 min. The product was then dispersed in deionized water and magnetically stirred in a glass beaker for 20 min. The suspension was ultrasonicated for 60 min in ice bath conditions, filtrated and vacuum dried to obtain *m*-GNPs (or *m*-CNTs). 

The calculated amounts of *m*-GNPs (0 wt%, 0.80 wt%, 2.35 wt% and 3.85 wt%) and *m*-CNTs (9.62 wt%) were dispersed in toluene at 2 mg/mL under the action of mechanical stirring; this was followed by sonication for 60 min. The *m*-GNPs and *m*-CNTs suspensions were subsequently mechanically stirred for 10 min and ultrasonicated for 30 min. Then CNT/GNP suspension was added to the EUG toluene solution; the mixture was then mechanically stirred for 30 min and ultrasonicated for 2 h. The mixture was precipitated using ethanol and then dried at 40 °C. The dried mixture was mixed with curing agents (ZnO, 4 phr; SA, 2 phr; sulfur, 2 phr; NOBS, 1.2 phr) on a two-roll mill at 65 °C. Then, the blends were cured into 1-mm-thick sheets under 150 °C at the vulcanization time. The CNT/GNP/EUG composites are referred to as ECGx, where x represents the content of GNPs added to the composites.

#### 2.2.2. Characterization

The morphology of the composites was investigated via scanning electron microscopy (SEM, SU8010, Hitachi Co., Ltd., Tokyo, Japan) and transmission electron microscopy (TEM, FEI Talos F200, Thermo Fisher Scientific Inc, Shanghai, China). The samples used for SEM were fractured in liquid nitrogen and had a surface coated with a thin gold layer. The TEM samples were ultramicrotomed at −100 °C to produce sections with a thickness in the range of 50–60 nm. Dynamic mechanical rheology measurements were performed using a rubber process analyzer (RPA-8000, Goteh Testing Machines Co., Ltd., Qingdao, China). A strain sweep was undertaken from 1% to 200% at 60 °C and 1 Hz. 

The electrical conductivity of the composites was measured using a four-probe conductometer (RTS-9, Guangzhou Four-Probes Technology Co. Ltd., Guangzhou, China). The EMI shielding performance of the composites was measured in the frequency range of 8.2–12.4 GHz (X band) at room temperature, using a vector network analyzer (E5071C, Agilent Technologies, Santa Clara, CA, USA); the test samples were of length 22.86 mm, width 10.16 mm and thickness 2.00 mm. The EMI performance parameters, including the SE total (*SE_T_*), SE reflection (*SE_R_*) and SE absorption (*SE_A_*), were calculated from the scattering parameters (*S*_11_ and *S*_21_).

(1) $$R={\left|S_{11}\right|}^{2}\;$$

(2) $$T={\left|S_{21}\right|}^{2}\;$$

(3) $$SE_{R}=-log_{10}\left(1-R\right)$$

(4) $$SE_{A}=-log_{10}\left(T/(1-R\right))$$

(5) $$SE_{T}=SE_{R}+SE_{A}\;$$

The tensile tests were carried out using an INSTRON 3365 testing machine (Instron Co., Ltd., Norwood, MA, USA) according to the standard ASTM D412 using a crosshead speed of 500 mm/min at 23 ± 2 °C. Cut the EUG/CNTs/GNPs composite sample into strips with a section width of 6 mm and thickness of 2 mm with a dumbbell cutter, and clamp both ends of each sample. For each data point, at least five specimens were tested, and an average value was taken. The thermal stability of the samples was measured via thermogravimetric analysis (TGA, Q50, TA Instruments, Rutherfordton, NC, USA) from 40 °C to 600 °C using a heating rate of 10 °C/min in an N_2_ atmosphere. Differential scanning calorimetric (DSC) measurements were obtained using a DSC-Q200 (TA Instruments, Rutherfordton, NC, USA) under N_2_ from −70 °C to 100 °C at a heating/cooling rate of 10 °C/min. The crystallinity of the EUG was determined, as

(6) $$\;X_{c}=\frac{\Delta H_{m}}{\Delta H_{m}^{*}}\times 100\%$$

where $\Delta H_{m}^{*}$ is the theoretical value of 100% crystallized EUG (125.6 J/g).

## 3. Results and Discussion

### 3.1. The Morphology of the CNT/GNP/EUG Composites

**Figure 1:**
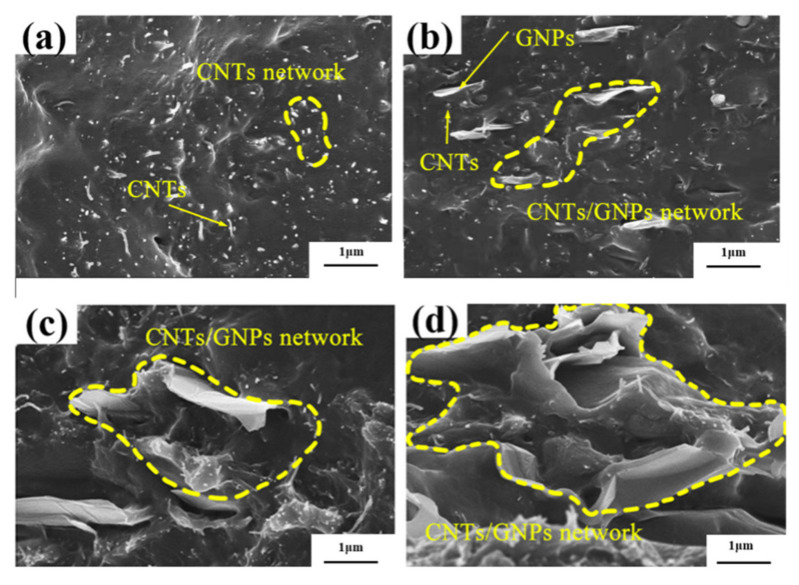
SEM images of CNT/GNP/EUG composites: (**a**) ECG0; (**b**) ECG1; (**c**) ECG2; (**d**) ECG3.

**Figure 2:**
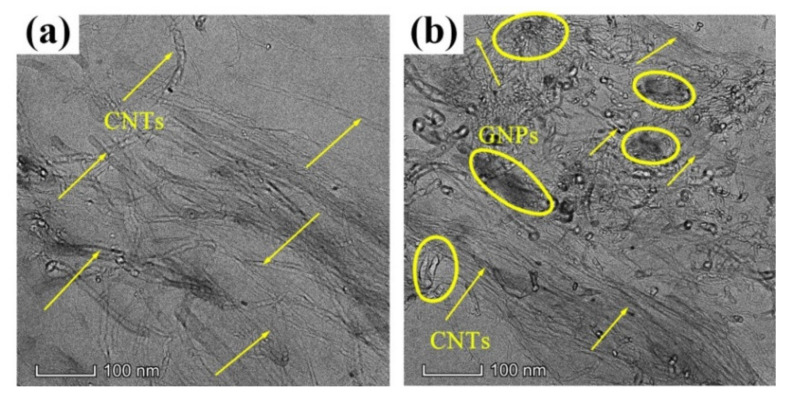
TEM images of CNT/GNP/EUG composites: (**a**) ECG0; (**b**) ECG3.

It is generally accepted that the properties of composite material are closely correlated with the dispersion of the filler material used. Figure 1 and Figure 2 show the SEM and TEM images of CNT/GNP/EUG composites. As shown in Figure 1a and Figure 2a, the CNTs are uniformly dispersed within the EUG matrix without obvious agglomeration or entanglement. As shown in Figure 1b–d and Figure 2b, the CNTs and GNPs are uniformly dispersed across the matrix after the shear force was applied during the open mill process. CNTs were observed to show an individual distribution state and are seen to be closely attached to the GNPs, further demonstrating that the GNPs lead to an efficient dispersion of the CNTs and implying that there exists a high-strength binding interaction between the GNPs and CNTs. Because of the π–π interaction between the CNTs and GNPs, GNPs can improve the dispersibility of CNTs and inhibit their re-aggregation due to their larger surface area and steric hindrance; rod-shaped CNTs can also reduce stacking effects of the GNPs [42]. Furthermore, with an increase in hybrid loading, the GNPs exhibit increased agglomeration; these were caused by the effect of the π–π bond between the GNPs and the strong Van der Waals force. Thus, the composites form a more complete CNT/GNP conductive network, which provides a favorable condition for the electrical conductivity of the composites.

**Figure 3:**
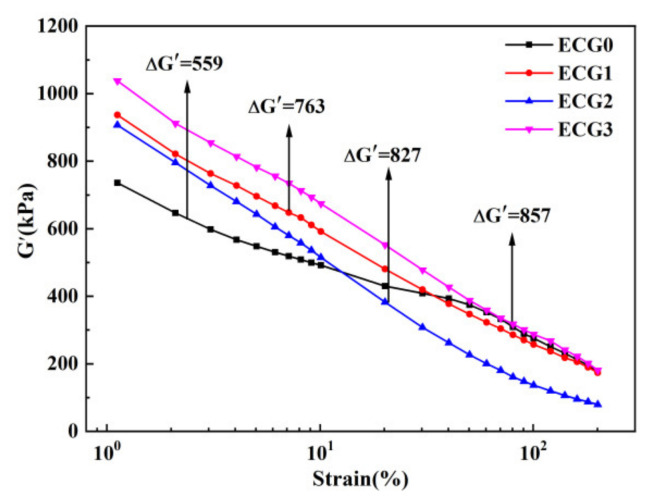
Storage modulus versus strain of CNT/GNP/EUG composites.

The Payne effect is widely used to characterize filler dispersion and filler networks. Figure 3 shows the storage modulus (G′) as a function of the strain for the CNT/GNP/EUG composites. G′ of the CNT/GNP/EUG composites is seen to decrease rapidly with increasing dynamic strain amplitudes; it is also seen to exhibit high sensitivity to strain, an indication of a typical Payne effect. The decrease in G′ is due to the destruction of filler networks. G′ increases significantly with increasing GNP loading of the CNT/GNP hybrids, demonstrating that denser filler networks are formed between CNTs and GNPs at higher CNT/GNP loading. The difference between G′ at 200% and G′ at 1% (referred to as ΔG′) also increases with the increase in the GNP loading; this phenomenon indicates an increase in the Payne effect. In summary, with increasing GNP loading within CNT/GNP hybrids, the filler networks possess a higher modulus and a higher sensitivity to strain. The strong filler networks also demonstrate that the CNT/GNP hybrids are highly dispersed within the EUG matrix. 

### 3.2. Electrical Conductivity and EMI Shielding Properties of the CNT/GNP/EUG Composites

**Figure 4:**
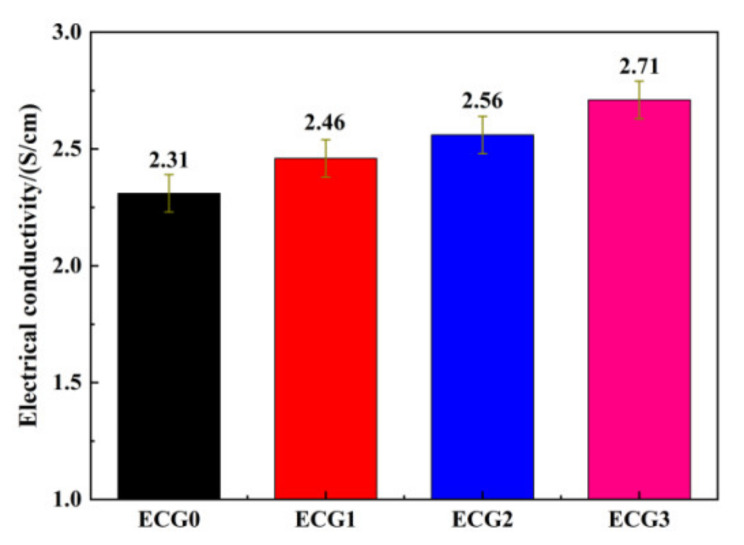
Electrical conductivity of CNT/GNP/EUG composites.

Electrical conductivity is a key factor that determines the EMI shielding properties of a given composite [43]. Materials with high electrical conductivity usually exhibit good EMI shielding properties. The volume electrical conductivity values of the CNT/GNP/EUG composites fabricated here are shown in Figure 4. CNTs are observed to be non-directionally dispersed and distributed but in a directional distribution in local microbeams, which results in the composites having anisotropic characteristics across different micro-domains; these properties have an effect on the electrical conductivity of the rubber. The electrical conductivity of CNTs/EUG composites with 9.62 wt% CNTs is seen to be around 2.31 S/cm. This result indicates that the CNTs in the composites form a conductive network at this CNT loading. The electrical conductivity of the CNT/GNP/EUG composites slightly increases with increasing of GNP loading within CNT/GNP hybrids. As shown in Figure 2, the CNT/GNP hybrids were well dispersed within the EUG matrix, which is conducive to the formation of perfect conductive networks and leads to an improved EMI shielding performance.

**Figure 5:**
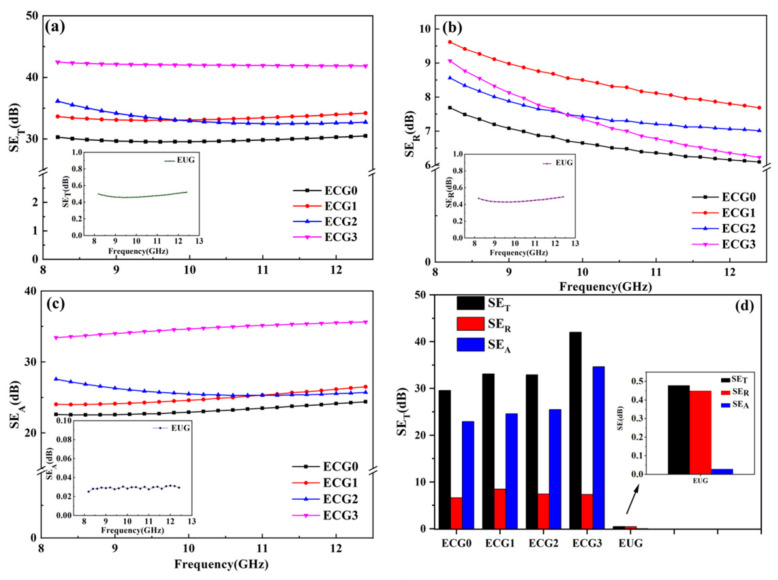
(**a**) EMI *SE_T_* (**b**) EMI *SE_R_*, (**c**) EMI *SE_A_* and (**d**) EMI *SE_T_* at 10 GHz of CNT/GNP/EUG composites.

The outstanding electrical properties endow CNT/GNP/EUG composites with high EMI shielding properties [44]. The EMI shielding properties of the CNT/GNP/EUG composites were assessed by measuring the values of *SE_T_*, *SE_R_* and *SE_A_*. Figure 5 shows the EMI *SE_T_*, *SE_R_* and *SE_A_* of the CNT/GNP/EUG composites in the X-band frequency range. The pure EUG exhibits a very small *SE_T_* value of less than 0.5 dB, indicating that EUG is almost completely transparent to the electromagnetic waves over the whole X-band frequency range. It can be seen from Figure 5a that the value of *SE_T_* of the CNT/GNP/EUG composites gradually increased from 30.29 dB to 42.49 dB with the increase in the GNPs loading within the CNT/GNP hybrids. The enhanced EMI shielding properties are attributed to the increased conductive networks created by the synergistic effect of GNPs and CNTs. 

**Figure 6:**
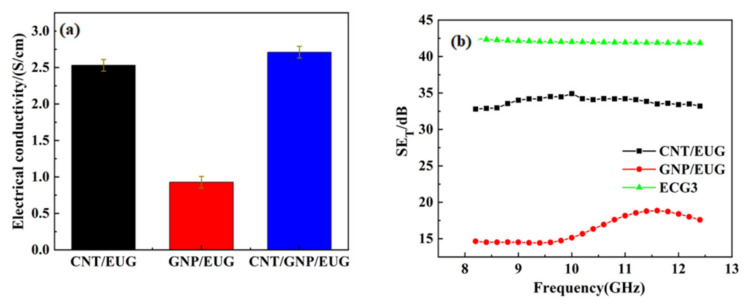
Comparison of (**a**) electrical conductivity and (**b**) EMI *SE_T_* of CNT/EUG composite (13.47 wt% CNT), GNP/EUG composite (13.47 wt% GNP) and CNT/GNP/EUG composite (9.62 wt% CNT and 3.85 wt% GNP, ECG3).

In order to state the synergistic effect of 2D GNPs and 1D CNTs, the same weight loading of GNP and CNT composites were fabricated, and the comparative results of electrical conductivity and EMI shielding efficiency are summarized in Figure 6. The electrical conductivity and EMI *SE_T_* of the CNT/GNP/EUG composite are all higher than CNT/EUG composite and GNP/EUG composite. As a 1D material, CNTs contact each other predominantly through point contacts. As a 2D material, GNPs contact each other via surface contacts. In CNT/GNP hybrids, GNPs function as the ‘spacers’ between the polymers, and the CNTs function as the ‘wires’ making up the conductive network. The combination of the two fillers creates much denser conductive networks than either filler used alone. Such perfect conductive networks permit the dissipation of electromagnetic waves through both reflection and absorption. Therefore, the CNT/GNP/EUG composites exhibit enhanced electrical conductivity and EMI shielding efficiency because of the synergistic effect of GNP and CNTs.

The maximum EMI SE_T_ value of the CNT/GNP/EUG composite is seen to be as high as ~42 dB, a value that meets the EMI shielding requirement for commercial products. Table 1 compares the results obtained in this work with other EMI shielding polymeric composites reported in previous work [17,18,20,28,30,31,32]; it is seen that the CNT/GNP/EUG composites created in this work exhibit superior EMI *SE_T_* compared with previously studied composites. The results indicate that this work provides a practical and effective method for the preparation of high-performance EUG-based EMI shielding composites. 

### 3.3. EMI Shielding Mechanism of the CNT/GNP/EUG Composites

In order to fully elucidate the EMI shielding mechanism of the CNT/GNP/EUG composites, the values of *SE_A_*, *SE_R_* and the relative values at 10 GHz were calculated and plotted in Figure 5b,c,d, respectively. The values of *SE_A_* and *SE_R_* of the CNT/GNP/EUG composites all increase with increases in GNP loading of the CNT/GNP hybrids. More importantly, the *SE_A_* values greatly increase with the GNP loading of the CNT/GNP hybrids. In all the CNT/GNP/EUG composites, the *SE_A_* values are much greater than the *SE_R_* values; this is because the conductive networks provide a sufficient number of interfaces that form multiple reflections and thus attenuate the incident electromagnetic waves [45]. For instance, the *SE_T_*, *SE_R_* and *SE_A_* of ECG3 are 42 dB, 7.4 dB and 34.6 dB, respectively. Furthermore, the *SE_A_* value that represents absorption makes up 82% of the *SE_T_* value, whereas the *SE_R_* value that represents reflection accounts for only 18% of the *SE_T_* value. Thus, the results of this work indicate that absorption is the predominant shielding mechanism of the CNT/GNP/EUG composites.

**Figure 7:**
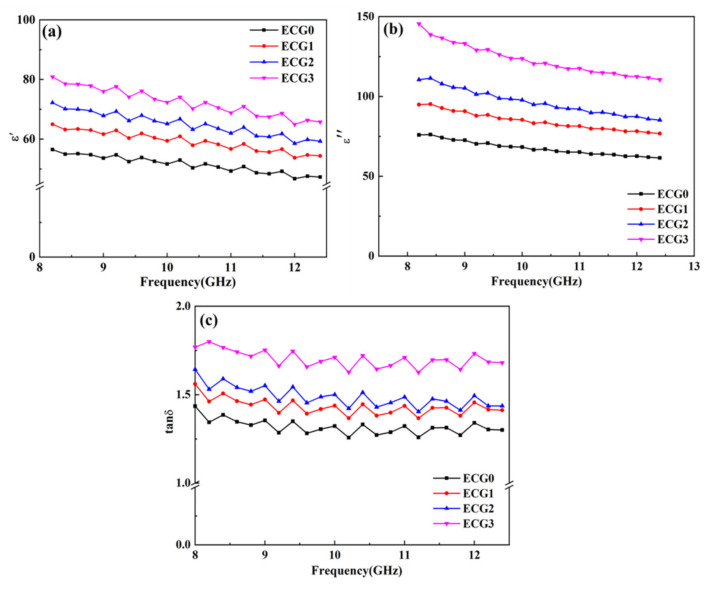
(**a**) *ε*’, (**b**) *ε*″ and (**c**) tan *δ_ε_* of CNT/GNP/EUG composites.

The complex permittivity of the CNT/GNP/EUG composites was also measured in order to understand their EM absorption properties better. Figure 7 shows the real part (*ε’*), the imaginary part (*ε″*) and the dielectric loss tangent (tan *δ_ε_* = *ε″*/*ε′*) in the X-band frequency range. The values of *ε’* and *ε″* represent the storage and loss capability of electric energy within a material, respectively, whereas tan *δ_ε_* is used to evaluate the dielectric loss capacity of a material. Both *ε’* and *ε″* decrease with increasing frequency; this decrease is related to the interface polarization [46]. It is noted that the values of *ε’* and *ε″* of the CNT/GNP/EUG composites increase with an increase in the GNP loading of the CNT/GNP hybrids. This is ascribed to the formation of perfect conducting networks by the CNTs and GNPs within the EUG matrix. The enhanced *ε’* value is predominantly due to the creation of more dipoles within the composite. The uniform distribution of the CNT/GNP hybrids without agglomeration can create many interfaces and hence induce polarization at the interfaces of CNT/GNP, CNT/EUG and GNP/EUG. Moreover, the introduction of more layers of GNPs with a large specific surface area induces an increase in the interface polarization [47]. The increase in the value of *ε″* with an increase in the GNP loading is attributed to the dielectric relaxation and electrical conductivity of the composites. The incorporation of GNPs enhances the electrical conductivity, resulting in high conduction loss. Furthermore, the presence of a high number of interfaces between the EUG matrix and the fillers results in further increases in the dielectric loss within the composite. The value of tan *δ_ε_* is significantly increased by increases in GNP loading, indicating a superior dielectric loss capacity. The enhanced value of tan *δ_ε_* represents significant dissipation of electrical energy and attenuation of wave absorption. 

**Figure 8:**
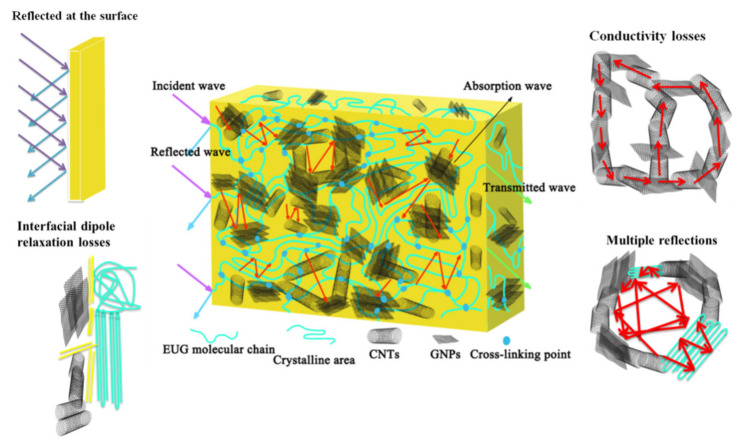
Proposed electromagnetic shielding mechanism for CNT/GNP/EUG composites.

Based on the preceding analysis, we established the shielding mechanism of the CNT/GNP/EUG composites, which is illustrated in Figure 8. When the EM waves reach the interfaces within the composite, a proportion of the EM waves are reflected at the surface of the CNT/GNP/EUG composite due to the impedance mismatch between the composites and the air, while a proportion of EM waves is absorbed by the CNT/GNP/EUG composite due to the conduction losses, multiple reflections from interfaces and the interfacial dipole relaxation losses. The CNT/GNP/EUG composites form more effective conductive networks due to the synergistic effect of 2D GNPs and 1D CNTs, thereby causing the attenuation of EM waves via Joule heating, which increases the EM absorption. Moreover, the numerous EUG/CNT, EUG/GNP and CNT/GNP interfaces facilitate substantial attenuation of the EM waves via multiple reflections and scattering. The interfacial polarizations also attenuate EM waves and enhance the EM wave absorption effect. Importantly, the boundary of crystalline–amorphous phases within EUG matrix can accumulate electrical charges, thus resulting in polarization loss, while the crystalline regions of the EUG matrix also increase the propagation of EM waves within the material and hence enhances EM absorption [48,49].

### 3.4. Crystallinity of the CNT/GNP/EUG Composites

**Figure 9:**
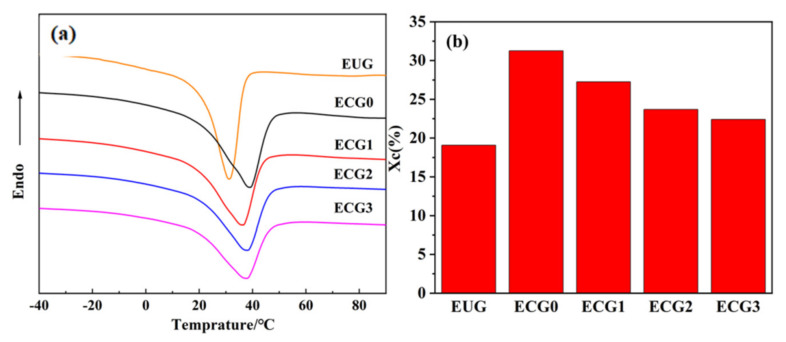
(**a**) DSC curves of CNT/GNP/EUG composites, (**b**) the crystallinity (Xc) of CNT/GNP/EUG composites.

Thermal and crystalline behaviors were characterized by the DSC curves, and the crystallinity of EUG was calculated. As shown in Figure 9a, all samples exhibit one peak upon heating curves, which corresponds to the melting peak of crystalline EUG. Compared with pure EUG, the melting temperature (*T_m_*) of the CNT/EUG composite shifts to a higher temperature, and the melting enthalpy (Δ*H_m_*) increases from −23.95 to −35.48 J/g. Accordingly, the crystallinity (*X_C_*) of the CNT/EUG composite also increases from 19.1% to 28.2%, implying that CNTs acted as the nucleating agent and promoted the crystallization of EUG segments. However, the *T_m_*, Δ*H_m_* and *X_C_* of CNT/GNP/EUG composite all decrease with the increase in GNP loading of the CNT/GNP hybrids. These results indicate that the EUG segmental mobility was restricted by strong networks formed by the synergistic effect of CNTs and GNPs. 

### 3.5. Thermal and Mechanical Properties of the CNT/GNP/EUG Composites

**Figure 10:**
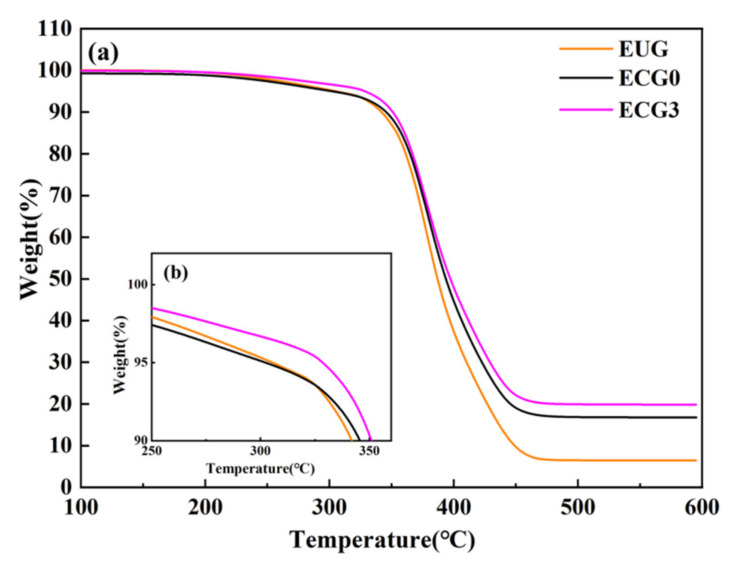
(**a**) Thermogravimetric curves of CNT/GNP/EUG composites. (**b**) Enlarged view of thermogravimetry at 100–90%.

Figure 10 depicts the TGA curves of the CNT/GNP/EUG composites; the related data are shown in Table 2. As shown in Figure 8, the thermal decomposition of CNT/GNP/EUG composites takes place in a single stage between 260 °C and 300 °C, predominantly due to the pyrolysis of the EUG macromolecules. The decomposition temperatures of the CNT/GNP/EUG composites with 5% and 30% weight loss (*T_5_*, *T_30_*) and the calculated heat resistance index (*T_HRI_*) are all higher than 303 °C, 410 °C and 180 °C, respectively. The value of *T_HRI_* of the CNT/GNP/EUG composites all increase with the increase in the GNP loading of the CNT/GNP hybrids. The value of *T_HRI_* for the CNT/GNP/EUG is 191 °C, which is 11 °C higher than that of pure EUG (180 °C). The reason for this increase is due to the CNT and GNP fillers possessing high heat resistance, which improves the thermal stability of the EUG matrix. 

**Figure 11:**
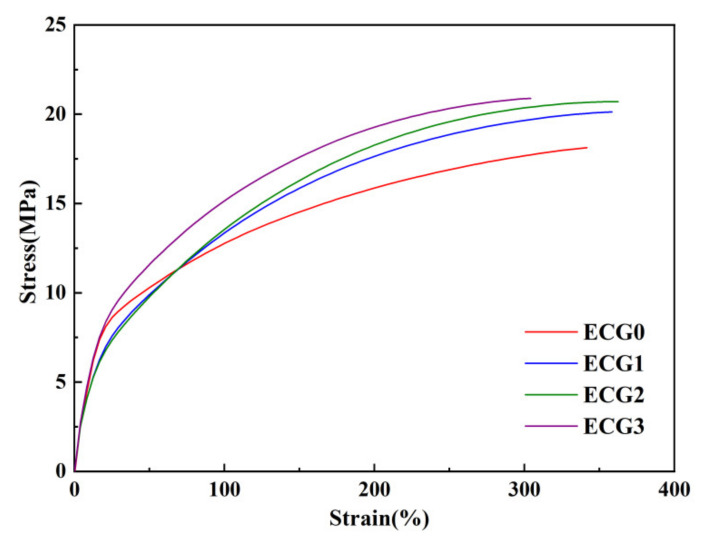
Stress-strain curves of CNT/GNP/EUG composites.

The mechanical properties of the CNT/GNP/EUG composites are shown in Figure 11. The tensile strength of the CNT/GNP/EUG composites is seen to increase with an increase in the GNP loading of the CNT/GNP hybrids. The elongation at break initially increases and subsequently decreases with increasing GNP loading of the composite. The incorporation of GNPs in the CNT/GNP/EUG composite improves the dispersion of CNTs, resulting in enhanced tensile strength and elongation at break. On the other hand, the introduction of more CNT/GNP hybrids increases the filler–filler and filler–polymer networks, which further reinforce the composites and resists the removal of polymer chains; this results in improved tensile strength and decreased elongation at break. The Young’s modulus of ECG0, ECG1, ECG2 and ECG3 are 0.05 GPa, 0.051 GPa, 0.052 GPa and 0.057 GPa, respectively. Besides, the shore A hardness of ECG0, ECG1, ECG2 and ECG3 are 79, 81, 82 and 84. The Young’s modulus and the hardness of the CNT/GNP/EUG composite show a slight increase with the increase in the CNT/GNP hybrids, an indication of the reinforcement of CNT/GNP hybrids. ECG3 exhibits a tensile strength of 20.9 MPa and elongation at a break of 304%. Therefore, the combination of the excellent electromagnetic shielding performance and high mechanical properties makes the as-prepared CNT/GNP/EUG composite appropriate for a wide range of applications.

## 4. Conclusions

In summary, we have successfully fabricated bio-based composites with a high EMI SE and attractive mechanical properties by combining a matrix of EUG with a crystalline structure and CNT/GNP hybrid structures used as conductive fillers. The synergistic effect of the CNT/GNP hybrid originates from the bridging of CNTs between GNPs, which facilitates the enhanced filler networks, electrical conductivity, EMI shielding properties and mechanical properties of the CNT/GNP/EUG composites. The maximum electrical conductivity and EMI SE values of CNT/GNP/EUG composites are observed to be up to 2.71 S/cm and 42.49 dB, respectively. Furthermore, the tensile strength of the CNT/GNP/EUG composites was found to increase with an increase in the GNP loading of the CNT/GNP hybrids. These results indicate that the method studied here provides a practical and effective route towards the creation of high-performance EUG-based EMI shielding composites.

## Figures and Tables

**Table 1:** Comparison of EMI shielding properties of CNT/GNP/EUG composites with other CPCs.

Materials	Carbon Filler Contents	Thickness (mm)	SE (dB)	Frequency Range	Ref.
CB/Silicone Rubber	15 phr	2.0	20	0.01–10,000 MHz	[17]
CF/MWCNT/PBT/PolyASA	8 vol %+2 vol %	2.0	33.7	8.2–12.4 GHz	[18]
CNT/PE	10 wt%	3.0	35	0.5–1.5 GHz	[20]
RGO/CB/PDMS	15 wt%+17 wt%	1.5	28	8.2–18 GHz	[28]
graphene/SBR	27.81 wt%	3.0	45	8–12 GHz	[30]
GN/NBR	4 wt%	2.0	77	1–12 GHz	[31]
CNTs/RGO/Epoxy	0.83 wt%+1.2 wt%	3	42	8.2–12.4 GHz	[32]
CNTs/GNPs/EUG	9.62 wt%+3.85 wt%	2.0	42.5	8.2–12.4 GHz	this work

PBT: Polyb; PBT: Polybutylene terephthalate; ASA: Acrylonitrile Styrene acrylate copolymer; PDMS: Polydimethylsiloxane; SBR: 1,3-butadiene polymer.

**Table 2:** Thermal characteristics data of CNT/GNP/EUG composites.

Samples	Weight Loss Temperature (°C)		T_Heat-resistance index_ (°C)
	T_d,5%_	T_d,30%_	
EUG	305	410	180
ECG0	303	423	184
ECG3	328	429	191

T_5_ and T_30_ correspond to the decomposition temperature of 5% and 30% weight loss, respectively.; T_Heat-resistance index_ = 0.49 ∗ [T_5_ + 0.6 ∗ (T_30_ − T_5_)].
